# Identification of Tobacco-Related Cancer Diagnoses among Individuals with Psychiatric Disorders: A Population-Based Matched Cohort Study Using a Competing Risks Approach from British Columbia

**DOI:** 10.3390/curroncol28060415

**Published:** 2021-11-24

**Authors:** Robert Olson, Mary McLay, Jeremy Hamm, Russell C. Callaghan

**Affiliations:** 1 Department of Radiation Oncology, BC Cancer—Prince George, 1215 Lethbridge Street, Prince George, BC V2M 7E9, Canada; 2 Department of Surgery, Division of Radiation Oncology and Developmental Radiotherapeutics, University of British Columbia, Vancouver, BC V6T 1Z4, Canada; 3 Northern Medical Program, University of Northern British Columbia, Prince George, BC V2N 4Z9, Canada; russell.callaghan@unbc.ca; 4 Department of Medicine, University of British Columbia, Vancouver, BC V6T 1Z4, Canada; mary.mclay@alumni.ubc.ca; 5 Department of Statistics, BC Cancer—Vancouver, Vancouver, BC V5Z 4E6, Canada; JHamm@bccancer.bc.ca

**Keywords:** cancer, psychiatric disorders, population-based, tobacco-related cancer

## Abstract

Background: Individuals with psychiatric disorders (PD) have a high prevalence of tobacco use. Therefore, we assessed the hazard of receiving a tobacco-related (TR) cancer diagnosis among individuals with PD. Methods: Several population-based provincial databases were used to identify individuals in BC diagnosed with depression, schizophrenia, bipolar disorder, anxiety disorders, or multiple PD between 1990 and 2013. A primary population proxy comparison group (appendicitis) was also identified and matched to the psychiatric cohort based on age at cohort entry, gender, year of cohort entry, and postal code. We linked individuals in the cohort and comparison groups with the BC Cancer Registry. Using a competing risks approach, we estimated the effect of having a PD on the risk of receiving a TR cancer diagnosis, in light of the competing risk of mortality. Results: In total, 165,289 patients were included. Individuals with depression (HR = 0.81; *p* < 0.01; 95% CI: 0.73–0.91), anxiety disorders (HR = 0.84; *p* = 0.02; 95% CI: 0.73–0.97), or multiple PD (HR = 0.74; *p* < 0.01; 95% CI: 0.66–0.83) had a statistically significant lower risk of a TR cancer diagnosis compared to the comparison group. Individuals with schizophrenia (HR = 0.86; *p* = 0.40; 95% CI: 0.62–1.21) or bipolar disorder (HR = 0.58; *p* = 0.12; 95% CI: 0.29–1.14), however, showed no evidence of a statistically significant difference from the comparison group. Interpretation: We found that individuals with depression, anxiety disorders, or multiple PD diagnoses had a significantly reduced risk of receiving a tobacco-related cancer diagnosis. These results were unexpected and could be explained by individuals with a PD having barriers to a cancer diagnosis rather than a true decreased incidence.

## 1. Introduction

Studies have shown that tobacco smoking is approximately two to four times more prevalent [1,2,3,4] and of higher intensity among individuals with psychiatric disorders (PD) compared to the general population. It is estimated that between 21% and 31% of patients with current tobacco dependence also have a current mood, anxiety, or psychiatric disorder [5]. Although smoking is on the decline overall, the decline among those with PD is significantly less [6]. Smoking rates range from 40–60% among individuals with depressive disorders, 45–88% for those with schizophrenia and related disorders [7], 55–70% for bipolar disorder, and 19.2–56% for anxiety disorders [4]. Despite these high rates, smoking cessation treatments are poorly integrated into psychiatric care, and little is known about the tobacco-related (TR) cancer trajectories in these populations.

Tobacco use is the leading preventable cause of disease and death worldwide, and it affects people with PD disproportionately. A meta-analysis of mortality among people with mental disorders found both higher risk of mortality and lower life expectancy than individuals without PD [8]. Previous studies have shown significantly elevated mortality for tobacco-related cardiovascular and respiratory diseases in PD populations [9]. However, there is conflicting literature on the tobacco-related cancer outcomes in these populations, most of which focus on schizophrenia [9]. Two studies found higher risk of cancer among individuals with PD, but were limited by looking at all cancers rather than each cancer individually and those associated with tobacco use, while the majority of individual cancers were not significantly more common [9].

The primary aim of this study, therefore, is to assess the hazard of receiving a TR cancer among individuals with PD in comparison to the general population. Our population-based cohort study addresses the gaps and limitations in the literature by using well-defined psychiatric cohorts to look specifically at tobacco-related cancer risk. We hypothesize that individuals with PD will be at higher risk of receiving a TR cancer than the general population.

## 2. Methods

### 2.1. Study Population: Data Sources

We conducted a retrospective cohort study of PD patients in BC using several population-based provincial databases through Population Data BC [10]. ICD codes from the Medical Services Plan (MSP) [11] and Discharge Abstracts Data (DAD) databases [12] and drug identification numbers (DIN) from PharmaNet/PharmaCare [13] were used to identify the psychiatric cohort. All individuals aged 13 to 85 in BC diagnosed with depression, schizophrenia and related disorders, bipolar disorder, anxiety disorders, or multiple PD between 1 January 1990 and 31 December 2013 were selected. A primary population proxy comparison group (appendicitis) was also identified using ICD codes from the MSP and DAD databases [14,15]. To be assigned to the appendicitis cohort, individuals could not have an ICD code for any PD in MSP or DAD databases during the study period.

### 2.2. Measurement of Outcomes

Individuals in both the cohort and comparison groups were linked with the BC Cancer Registry (BCCR) database [16], which captures all incident cases of cancer in the province. We identified individuals in the psychiatric and appendicitis cohorts who subsequently developed a TR cancer using the Surgeon General’s Report: The Health Consequences of Smoking—50 Years of Progress [17] and ICD-9 and ICD-10 definitions from the Centers for Disease Control and Prevention’s (CDC) Smoking-Attributable Mortality, Morbidity, and Economic Costs (SAMMEC) approach [17,18]. The following twelve cancers shown to be causally linked to smoking were selected: oral cavity and pharynx; larynx; esophagus; lung, bronchus and trachea; acute myeloid leukemia (AML); stomach; liver; pancreas; kidney and renal pelvis; cervix; urinary bladder; colorectal. The cohorts were also linked with the Vital Statistics Deaths database, where all deaths of BC residents are reported.

### 2.3. Statistical Methods

Descriptive statistics were performed on patient characteristics for all psychiatric and appendicitis patients. The number of patients diagnosed with PD in the study period greatly outnumbered patients diagnosed with appendicitis; therefore, a 3:1 greedy propensity score matching algorithm was applied to match the PD cohort to the appendicitis group. The cohorts were matched on age at diagnosis, gender, year of hospital admission, and 3-digit postal code (FSA used as a proxy for socioeconomic information). Age and admission year were given a ±2 year window to match on, while gender and FSA were matched directly.

A competing risk analysis was used to estimate the hazards of receiving a TR cancer. Follow-up time was calculated from the date of PD or appendicitis diagnosis to cancer diagnosis; if a patient did not develop a TR cancer, the study end date of 31 December 2014 was used. Using a competing risks approach, stratifying the variables used in the matching process, we then estimated the effect of having a PD on the risk of receiving a TR cancer in light of the competing risk of mortality. Follow-up time was calculated as the time from PD or appendicitis diagnosis to TR cancer or death, or 31 December 2014 if neither event occurred.

Since our results were contrary to our hypothesis (see below), we subsequently assessed the overall survival (OS) rates in both cohorts to ascertain whether early death in the PD cohort could possibly explain our results. OS rates were estimated using the Kaplan–Meier method and survival curves. Survival rates were compared among the comparison group and PD cohort using the log-rank test. Survival analyses were performed using a Cox regression and also stratified on the variables used in the matching process, to assess PD and appendicitis group differences in survival.

For survival analyses, time was calculated from the date of PD or appendicitis diagnosis until death. If subjects were still alive at the end of the study period, the censor time was calculated as the time from diagnosis until 31 December 2014, since all British Columbian deaths were accurate up to this date at the time of the analysis.

All tests were two-sided, with a *p*-value less than or equal to 0.05 considered to be significant. Analyses were conducted using Statistical Package for Social Sciences software version 23.0 (SPSS, Chicago, IL, USA) and SAS computer statistical package (Version 9.4; SAS Institute, Cary, NC, USA). This study received approval from the joint University of British Columbia and BC Cancer Agency Research Ethics Board.

## 3. Results

### 3.1. Clinical Characteristics

Between 1990 and 2013, 1,567,920 patients were diagnosed with a PD and 41,940 patients were diagnosed with appendicitis in BC. Of these patients, 123,752 individuals diagnosed with a PD were matched to 41,537 from the comparison. The prevalence of each PD in the cohort during this time was 42% depression, 2% schizophrenia and related disorders, 0.5% bipolar disorder, 17% anxiety disorders, and 39% multiple PD. Table 1 presents the patient demographics and follow-up information for the matched cohort.

### 3.2. Survival Analyses

**Figure 1:**
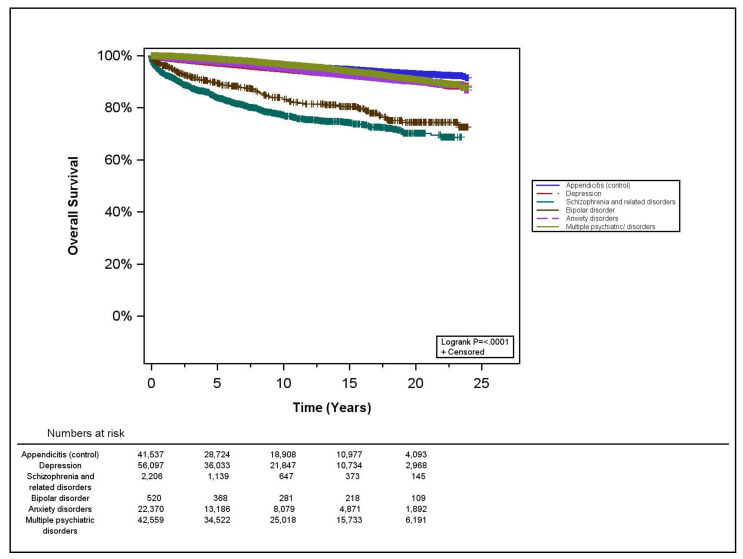
Kaplan–Meier estimates of overall survival by psychiatric disorder.

In order to assess whether death should be treated as a competing risk, we first performed our survival analyses. Individuals with PD were 1.37 times more likely to die during the study period than appendicitis patients (95% CI: 1.28–1.48). In our cohort, individuals with schizophrenia and bipolar disorder had the highest percentage of deaths at 19%, while 4% of anxiety patients and 5% of depression patients died during the study (compared to 4% of appendicitis patients) (Table 1). OS was significantly worse among the psychiatric cohorts (*p* < 0.001) (Figure 1). The 10-year OS rates were 97% for appendicitis, 95% for depression, 79% for schizophrenia, 84% for bipolar disorder, 96% for anxiety disorders, and 97% for multiple PD (*p* < 0.001). Full 5-, 10-, 15- and 20-year OS information is provided in supplementary Table S1. Table 2 presents the Cox regression analysis assessing the impacts of PD diagnosis on survival. All PD showed significantly worse survival compared to the appendicitis group (HR range = 1.09–6.52; *p* < 0.001) (Table 2).

### 3.3. Tobacco-Related (TR) Cancer Analysis

**Figure 2:**
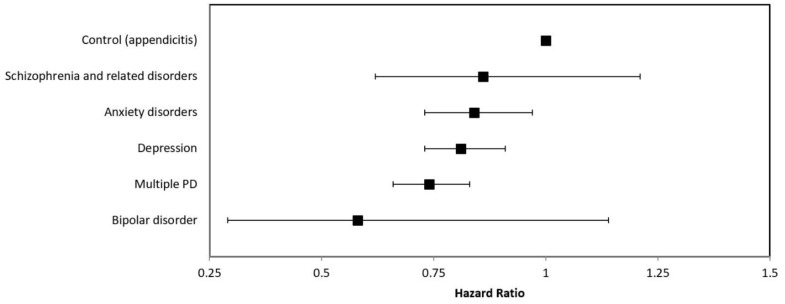
Competing risk estimates of the effect of having a psychiatric diagnosis on the risk of developing a tobacco-related cancer.

Individuals with PD had a 22% lower risk of TR cancer diagnosis than individuals with appendicitis (HR = 0.78, 95% CI: 0.71–0.85). When death was treated as a competing risk, patients with a PD were still less likely to be diagnosed with a TR cancer (Figure 2; Table 3). Individuals diagnosed with depression (HR = 0.81, 95% CI: 0.73–0.91), anxiety disorders (HR = 0.84, 95% CI: 0.73–0.97), or multiple PD (HR = 0.74, 95% CI: 0.66–0.83) were all found to have statistically significant lower hazard of TR cancer diagnosis than the comparison group. The hazards of TR cancer diagnosis among individuals diagnosed with schizophrenia and related disorders (HR = 0.86; 95% CI: 0.62–1.21) or bipolar disorder (HR = 0.58, 95% CI: 0.29–1.14) were not significantly different from the comparison group.

### 3.4. Interpretation

Contrary to our hypothesis, the results of this population-based cohort study suggest that individuals with PD do not have a higher risk of receiving a diagnosis of a tobacco-related cancer. In fact, individuals with depression, anxiety disorders, or multiple PD diagnoses have significantly reduced risk of receiving a TR cancer diagnosis compared to our population proxy comparison group. These results are surprising and counter-intuitive. We believe it is unlikely that there is a causal relationship between having a PD diagnosis and a decreased incidence of developing cancer. Therefore, we hypothesize that instead of there being a true decreased incidence of cancer, individuals with PD diagnosis have increased barriers to receiving a correct cancer diagnosis.

Before discussing the hypothesis that our findings show that PD patients have a decreased rate of cancer diagnosis rather than a decreased hazard of a true diagnosis, we wish to comment on the role of early mortality in PD patients. Our finding that patients with PD had inferior mortality outcomes in comparison to a general population proxy comparison group (Table 1) is consistent with the previously published literature [8,19]. A meta-analysis of mortality among people with mental disorders found a higher risk of mortality and lower life expectancy, a pooled relative risk of mortality of 2.22, and a median years of potential life lost score of 10 years [8]. The well described early mortality risk associated with having a PD likely at least partially describes the lower incidence of TR cancer. However, the results from our competing risk model account for this, and there is still no sign of an increased risk of TR cancer. Therefore, early mortality from PD cannot fully explain our results of a decreased hazard of receiving a cancer diagnosis. As hypothesized above, this may not be indicative of a true decreased hazard of cancer incidence.

The potential mechanisms for patients with PD having a decreased diagnosis of cancer, even if they have a similar or increased actual incidence, are broad and potentially concerning. It is plausible that the PD patients have a lower detection rate of clinically less meaningful cancers because of lower participation in screening and appointments with their family physicians (e.g., thyroid cancer, ductal carcinoma in situ, low risk prostate cancer); however, our study specifically limited the analysis to TR cancers, the majority of which are clinically significant cancers that become symptomatic (e.g., lung, esophagus, oral cavity) [20,21,22]. Even more concerning, it is plausible that PD patients did develop similar or higher rates of clinically meaningful cancers, yet these were also inappropriately detected at a lower rate. This could be the result of the stigma that PD patients experience, which could in turn lead to lower healthcare utilization and appropriate treatment for cancers that could be cured or palliated [21]. Unfortunately, our dataset cannot assess this.

Although we believe it to be far less likely, it could be hypothesized that there is a true decreased incidence of TR cancers in patients with PD. It is difficult to hypothesize a biologically plausible reason for the decreased incidence of TR cancers in patients with depression, anxiety, or multiple PD diagnoses, especially within the limits of these datasets. Future work could explore other potential explanations for this relationship, such as unknown cancer chemopreventative effect from psychiatric medical usage, although we do not know of any biological rational to pursue this specific relationship. Researchers who can hypothesize other biologically plausible relationships are encouraged to pursue future research.

This study should be interpreted within the context of its strengths and limitations. The study is limited by a lack of data on tobacco use in each cohort, meaning we cannot confirm that our PD patient population in BC has a higher tobacco use rate than our general population comparison group. However, given it is population-based, the use of tobacco should match the general population, free from the potential selection bias present in other methodological approaches. Our study is also strengthened by the provincial PopDataBC population-based data linkages of inpatient data, while the ability to link death data would complete a competing risk analysis. Furthermore, the large sample has sufficient power to identify differences in incidence amongst groups. Our study is also the first oncology study to use individuals hospitalized with appendicitis related conditions as a population proxy comparison group, which decreases potential biases in the estimations of hazard ratios, as described elsewhere [14], while also having the advantage of not being related to socioeconomic status or PD diagnoses [15,23,24,25].

## 4. Conclusions

This large population-based cohort study has identified a novel decreased hazard of being diagnosed with a TR cancer in individuals with depression, anxiety disorders, or multiple PD diagnoses. These results were unexpected, and we hypothesize they could be explained by individuals with a PD having increased barriers to a cancer diagnosis rather than a true decreased incidence. Most concerning, our limitation of the analysis of TR cancers, which are usually clinically meaningful, such as lung and esophageal cancers, suggests that PD stigma may prevent these patients from receiving diagnosis and care. Further research is needed to assess this hypothesis or to look for biologically plausible reasons why PD patients may have a true decreased incidence of TR cancer. A focus on lung cancer specifically is warranted.

## Figures and Tables

**Table 1:** Matched cohort demographics and follow-up information, comparing all psychiatric disorders and psychiatric disorder sub-cohorts to the appendicitis control group.

Characteristic	Control	A		B					
	(Appendicitis) *n* = 41,537	All PD *n* = 123,752	*p*-Value	Depression *n* = 56,097	Schizophrenia *n* = 2206	Bipolar *n* = 520	Anxiety *n* = 22,370	Multiple PD *n* = 42,559	*p*-Value
Median age at diagnosis in years (range)	29 (13–85)	30 (13–85)	0.29	30 (13–85)	33 (13–85)	29 (13–85)	31 (13–85)	28 (13–85)	<0.001
Age, *n* (%)									
13–25	17,105 (41%)	50,600 (41%)	0.02	22,251 (40%)	815 (37%)	228 (44%)	8262 (37%)	19,044 (45%)	<0.001
26–35	8523 (21%)	25,493 (21%)	0.95	11,326 (20%)	343 (16%)	89 (17%)	4680 (21%)	9055 (21%)	0.57
36–45	6216 (15%)	18,644 (15%)	0.92	8682 (16%)	221 (10%)	57 (11%)	3429 (15%)	6255 (15%)	0.24
46–55	4303 (10%)	12,885 (10%)	0.92	6304 (11%)	179 (8%)	43 (8%)	2488 (11%)	3871 (9%)	0.01
56–65	2766 (7%)	8257 (7%)	0.58	3961 (7%)	160 (7%)	40 (8%)	1874 (8%)	2222 (5%)	<0.001
66–75	1689 (4%)	5075 (4%)	0.63	2287 (4%)	215 (10%)	31 (6%)	1141 (5%)	1401 (3%)	0.001
76–85	935 (2%)	2798 (2%)	0.98	1286 (2%)	273 (12%)	32 (6%)	496 (2%)	711 (2%)	0.06
Male, *n* (%)	25,457 (61%)	75,596 (61%)	0.47	34,699 (62%)	1704 (77%)	351 (68%)	14,114 (63%)	24,728 (58%)	<0.001
Mean years of follow-up (to cancer)	9.9	9.8	0.06	8.7	7.4	11.6	8.5	12.0	<0.001
Developed TR cancer during study period, *n* (%)	730 (2%)	1689 (1%)	<0.001	721 (1%)	55 (3%)	11 (2%)	312 (1%)	590 (1%)	<0.001
Mean years of follow-up (to death)	10.9	10.8	0.003	9.7	8.2	12.5	9.5	13.0	<0.001
Died during study period, *n* (%)	1577 (4%)	6329 (5%)	<0.001	2696 (5%)	416 (19%)	96 (19%)	993 (4%)	2128 (5%)	<0.001
Total person-years of follow-up	409 251	1 210 346	-	487 950	16 285	6044	189 820	510 247	-

Note: 123,752 patients with PD were matched (3:1) to 41,537 appendicitis patients. All PD = any psychiatric disorder diagnosis; schizophrenia = schizophrenia and related diseases; bipolar = bipolar disorder; anxiety = anxiety disorders; multiple PD = diagnosis with 2 or more PD on same day.

**Table 2:** Cox regression model used to estimate the effects of having a PD on survival.

Cancer-Specific Survival		Overall Survival	
	Hazard Ratio	95% Confidence Interval	*p*-Value
Control (appendicitis)	1	reference	
Depression	1.41	1.33–1.50	<0.001
Schizophrenia and related disorders	6.47	5.81–7.21	<0.001
Bipolar disorder	4.25	3.46–5.22	<0.001
Anxiety disorders	1.33	1.23–1.44	<0.001
Multiple PD	1.11	1.04–1.18	<0.001

**Table 3:** Competing risk model used to estimate the effect of having a PD on the risk of developing a TR cancer in light of the competing risk of mortality.

	Hazard Ratio	95% Confidence Interval	*p*-Value
Control (appendicitis)	1	reference	
Depression	0.81	0.73–0.91	<0.01
Schizophrenia and related disorders	0.86	0.62–1.21	0.40
Bipolar disorder	0.58	0.29–1.14	0.12
Anxiety disorders	0.84	0.73–0.97	0.02
Multiple PD	0.74	0.66–0.83	<0.01

## Data Availability

The data are not available because of restrictions by Population Data BC.

## Supplementary Materials

The following are available online at https://www.mdpi.com/article/10.3390/curroncol28060415/s1, Supplementary Table S1: overall survival estimates by psychiatric disorder.
