# Supplementary material for: Identification of Tobacco-Related Cancer Diagnoses among Individuals with Psychiatric Disorders: A Population-Based Matched Cohort Study Using a Competing Risks Approach from British Columbia
Source: Curr Oncol. 2021 Nov 24;28(6):4953–60. doi: 10.3390/curroncol28060415 (PMC8628717; doi:10.3390/curroncol28060415)
Supplement: Supplementary file 1 [file curroncol-28-00415-s001.zip › curroncol-1450014-supplementary.pdf]

Current Oncology

MDPI

Article

Identification of Tobacco-Related Cancer Diagnoses among Individuals with Psychiatric Disorders: A Population-Based Matched Cohort Study Using a Competing Risks Approach from British Columbia

Robert Olson [1,2,*], Mary McLay [1,2], Jeremy Hamm [3] and Russell Callaghan [4]

Supplementary Table S1. Overall Survival estimate by psychiatric disorder.

	5-year OS	10-year OS	15-year OS	20-year OS	*p*-value
Control (Appendicitis)	98.0%	96.5%	95.0%	93.6%	< 0.001
Depression	97.3%	95.2%	93.0%	91.2%	
Schizophrenia and related disorders	84.8%	78.7%	75.9%	72.5%	
Bipolar disorder	89.9%	83.8%	81.1%	75.6%	
Anxiety disorders	97.7%	95.6%	93.0%	91.0%	
Multiple PD	98.8%	96.8%	94.4%	91.7%	
All PD	97.6%	95.5%	93.1%	90.8%	< 0.001
